# VIP activates primordial follicles of rat through ERK-mTOR pathway in tissue culture

**DOI:** 10.1530/REP-18-0466

**Published:** 2019-02-26

**Authors:** Song Li, Qi Fan, Yanqiu Xie, Haiyan Lin, Qi Qiu, Yihua Liang, Qingxue Zhang

**Affiliations:** 1 Department of Obstetrics and Gynaecology, IVF Centre, Sun Yat-Sen Memorial Hospital, Sun Yat-Sen University, Guangzhou, China; 2 Guangdong Provincial Key Laboratory of Malignant Tumour Epigenetics and Gene Regulation, Sun Yat-Sen Memorial Hospital, Sun Yat-Sen University, Guangzhou, China; 3 Department of Obstetrics and Gynaecology, IVF Centre, Guangdong General Hospital, Guangzhou, China

## Abstract

*In vitro* activation of primordial follicles is becoming more essential in assisted reproductive technologies. Vasoactive intestinal peptide (VIP) is one of the members of the neurotrophin family which has demonstrated to have an impact on follicle development in recent years. This study aims to investigate the effect of VIP on the activation of primordial follicles in neonatal rat in an *in vitro* culture system and to determine the relevant molecular mechanism of their activation. Ovaries of 4-day-old rats were examined for the expression of VIP receptors and were cultured in mediums containing VIP with or without inhibitors of the ERK–mTOR signalling pathway. They were then collected for histological analysis or measurement of the molecular expression of this pathway. The receptors of VIP were found in granular cells and oocytes of primordial and early-growing follicles in neonatal ovary. The ratio of growing follicle increased in the presence VIP at different concentrations, with the highest level of increase being observed in the 10^−7^ mol/L VIP-treated group. The ratio of PCNA-positive granular cells was also increased, while that of the apoptotic oocytes were decreased, and protein analysis showed increased phosphorylation of ERK1/2, mTOR and RPS6 in the VIP-treated group. However, the effect of VIP on the activation of primordial follicle became insignificant with the addition of MEK inhibitor (U0126) or mTORC1 inhibitor (rapamycin). This study indicated that VIP could activate neonatal rat primordial follicle through the ERK-mTOR signalling pathway, suggesting a strategy for *in vitro* primordial follicle recruitment.

## Introduction

Cryopreservation of ovarian tissue is an important way to preserve fertility in women who are facing fertility-threatening diseases or treatments (Ladanyi *et al.* 2017, Kim *et al.* 2018). However, re-transplantation of ovarian tissue may carry the risk of malignancy relapse. Poor surgical procedure or limited ovarian tissue might also cause infertility after re-transplantation (Kim *et al.* 2018). To solve this problem, researchers are progressively exploring suitable ways to obtain matured oocytes through *in vitro* activation (IVA) of primordial follicle. Some researchers have obtained ovary tissue from patients with ovarian insufficiency for IVA that involves Hippo signalling disruption and protein kinase B (AKT) stimulation, followed by auto-transplantation and assisted reproductive technologies. Even though live birth was achieved after re-transplantation of the cultured ovarian tissue, the low success rate made it necessary for new IVA strategy to be developed (Suzuki *et al.* 2015, Zhai *et al.* 2016). In view of the complexity of primordial follicle activation, the application of inhibitor or stimulator of a key signalling pathway may not be sufficient. According to literature, a series of growth factors are involved in follicle development (Zhang & Liu 2015), but how these factors influence the activation of primordial follicles remains unclear. Therefore, revealing the functions and mechanism of these factors may be of great benefit to primordial follicle development.

Vasoactive intestinal peptide (VIP), which is mainly produced by the central and peripheral nervous system as a neuroendocrine hormone, a neurotransmitter or a cytokine, has many important biological effects including relaxing the blood vessels, promoting gastrointestinal peristalsis, regulating the immune system and protecting the nervous system (Onoue *et al.* 2008). In recent years, VIP was found to be associated with the ovary function (Streiter *et al.* 2016). The mRNA transcripts or protein of VIP and its receptors, VPAC1 and VPAC2, were identified in the ovaries of some mammals (Hulshof *et al.* 1994, Barberi *et al.* 2007, Gabbay-Benziv *et al.* 2012, Bukowski & Wąsowicz 2015). Many studies have since showed that VIP might influence the development of preantral and antral follicles (George & Ojeda 1987, Mayerhofer *et al.* 1997, Cecconi *et al.* 2004). We have previously demonstrated that VIP could promote the development of neonatal rat ovary during *in vitro* culture (Chen *et al.* 2013). However, the expression and localization of proteins for VIP and its receptors in neonatal rat ovary, and the exact mechanism by which VIP influences the recruitment of primordial follicle has not yet been revealed.

VIP phosphorylates various proteins mainly through the cyclic adenosine monophosphate (cAMP)-protein kinase A (PKA) signalling pathway after binding to VPAC and produces physiological effects, such as vasodilation and immune regulation, promoting digestion (Couvineau & Laburthe 2012). Mitogen-activated protein kinase (MAPK), one of the downstream proteins of PKA, has been implicated as a key regulator of cell proliferation and differentiation (Pearson *et al.* 2001). One of the three major classes of MAPKs in mammals is mitogen-activated protein kinases 3 and 1(MAPK3/1) (also known as extracellular signal-regulated kinases 1 and 2 (ERK1/2)), of which phosphorylation is mediated by a MAPK kinase (MAPKK, otherwise known as MAPK-ERK kinase 1, MEK1) (Davis 2000). Previous studies have shown that ERK1/2 took part in the activation of primordial follicles (Zhao *et al.* 2018). Zhang *et al.* recently found that the recruitment of primordial follicles is triggered by mTORC1 activation in pregranular cells in a gene-knockout rat model (Zhang *et al.* 2014). A study has suggested that ERK1/2 could regulate the activity of mTORC1 in the activation of primordial follicles (Zhao *et al.* 2018); therefore, we hypothesize that VIP could promote the recruitment of primordial follicles through the ERK-mTORC1 signalling pathway.

The activation of primordial follicles is the first step in follicle development to produce a viable oocyte. This highlights the importance of elucidating the regulatory mechanism involved in order to provide theoretical support for preserving female fertility and treatment of infertility. In this study, we examined the expression of VIP and its receptors in the neonatal rat ovary and revealed the mechanism of how VIP affected the development of primordial follicle.

## Materials and methods

### Animals and reagents

Four-day-old *Rattus norvegicus* (Sprague–Dawley) female rats were obtained from the Centre of Experimental Animals, Sun Yat-Sen University. They were housed under controlled conditions of temperature (20–26°C), relative humidity (35–75%) and photoperiod (12L: 12D), with free access to food and tap water and housed with their mother. Experimental protocols were approved by the Institutional Animal Care and Use Committee (IACUC) of Sun Yat-Sen University.

The reagents used in the present study were obtained from the following sources and handled as follows: vasoactive intestinal peptide (VIP) (1911) from Tocris Bioscience (Bristol, UK) was dissolved to 3.0067 × 10^−4^ mol/L in water; VIP_6–28_ (V4508) from Sigma-Aldrich was dissolved to 1.775 × 10^−3^ mol/L in water; U0126 (#9903) from Cell Signaling Technology was dissolved to 0.01 mol/L in DMSO; Rapamycin (53123-88-9) from Gene Operation (Michigan, USA) was soluble in DMSO at 0.055 mol/L. All reagents mentioned above were stored under −20°C conditions and diluted to suitable concentration in the culture medium (see below).

### Ovary culture

We had previously successfully established an *in vitro* ovary culture system (Chen *et al.* 2013). Ovaries were dissected from 4-day-old female rats using small dissecting instruments and isolated in phosphate-buffered saline (PBS, Gibco BRL) under stereomicroscope (SteREO Discovery V.12, ZEISS). The ovaries were then washed three times with warmed sterile PBS. Whole ovaries were randomly distributed into groups and two ovaries were placed in a Millicell-CM filter insert each (pore size, 0.4 μm, 3413, Corning) floating on the culture medium in individual wells of a 24-well culture dish (Corning), which was previously equilibrated for 30 min with 600 μL of culture medium, ensuring that the ovaries were completely covered by medium. The plates were incubated at 37°C with 5% CO_2_. The medium consisted of a mixture of Dulbecco’s modified Eagle medium plus F-12 medium (1:1 v:v; Gibco BRL) containing 0.05% bovine serum albumin (Sigma-Aldrich), 1% ITS-X (effective concentrations: 10 mg/mL of insulin, 5.5 mg/mL of transferrin, and 6.7 ng/mL of sodium selenite; Gibco BRL), 0.05 mg/mL of L-ascorbic acid (Sigma-Aldrich), antibiotics (100 U/mL of penicillin, 100 μg/mL of streptomycin and 5 μg/mL of Fungizone (amphotericin B), all from Gibco BRL). The culture medium was replaced with fresh medium daily. After a 3-day *in vitro* culture, ovaries were immediately placed into 4% paraformaldehyde (PFA, Leagene, Beijing, China) for histological follicle assessment or stored in −80°C for western blotting or qPCR.

### Ovarian histomorphology

The ovaries were fixed in 4% PFA for 24 h, and then dehydrated, paraffin-embedded and cut into 5 μm sections. The sections were rehydrated and stained with hematoxylin and eosin (HE, Leagene, Beijing, China) for morphological observation and differential follicle counts. Follicle counting was performed on every fifth section of the ovaries by two independent investigators who were blinded to the experiment. Follicle stages were identified according to the following definitions: the primordial follicle is counted when the nucleus is surrounded by a flat layer of pre-granular cells; primary follicle is an oocyte encircled by a single layer of cuboidal granular cells; secondary follicle has at least two layers of cuboidal granulosa cells without antrum and the antral follicle has an antrum. Both primary follicles and secondary follicles are grouped as early-growing follicles. Occasionally, follicles may consist of both cuboidal and flat squamous granulosa cells, which indicate an intermediate stage between the primordial and primary stages. In these cases, stage identification was decided according to the type of predominant granular cells (Chen *et al.* 2013).

### Immunohistochemistry and immunofluorescence

After deparaffinization and rehydration, the sections were incubated in citrate buffer (pH 6.0, Wuhan Servicebio Technology Co., Ltd., China) and were microwaved for 10 min, and then immersed in 3% H_2_O_2_ for 10 min at room temperature to block endogenous peroxidase activity. Nonspecific binding was blocked with 10% normal goat serum (Beijing Biosynthesis Biotechnology Co., Ltd. (Bioss), Beijing, China) for 1 h at 37°C. The sections were incubated with the primary antibody (anti-VIP (1:200, ab8556), anti-VPAC1 (1:200, ab245743), anti-VPAC2 (1:200, ab216630) and anti-PCNA (1:400, ab92552)) (Abcam) for 24 h at 4°C followed by room temperature placement for 30 min. For immunohistochemistry, horseradish peroxidase (HRP)-combined secondary antibodies (Zhongshan Golden Bridge Bio-technology, Beijing, China) were added for a 30-min reaction at 37°C, followed by diaminobenzidine (DAB, Zhongshan Golden Bridge Bio-technology) staining. The sections were counterstained with hematoxylin and then dehydrated and mounted with neutral balsam. For immunofluorescence, Cy3-conjugated secondary antibodies (Jackson ImmunoResearch Laboratories, Inc.) were added at 37°C for 1 h, followed by 4,6-diamidino-2-phenyiindole (DAPI, BestBio, Shanghai, China) staining for 5 min. The sections were washed three times (5 min each) with PBS after each of the above procedure. At the same time, a negative control was treated by replacing primary antibodies with PBS. Finally, the sections were observed and photographed under the microscope.

### TUNEL assay

In Situ Cell Death Detection Kit-POD (Roche), a commercial kit of TUNEL assay, was employed to evaluate cell apoptosis of follicles. After dewaxing and rehydrating, the sections were incubated in citrate buffer (pH 6.0) and heated by microwave for 10 min, and then rinsed twice with PBS. A 50 µL TUNEL working solution per sample was mixed with label solution and enzyme solution, while the negative control used label solution. The sections were incubated at 37°C for 60 min in the dark, followed by rinsing twice with PBS. Then the nuclei were counterstained with DAPI for 10 min and washed three times with PBS (5 min each). Finally, the fluorescence of the sections was photographed and analysed by fluorescence microscope (ZEISS).

### Western blotting

Ovaries were placed in RIPA lysis buffer (Beyotime Institute of Biotechnology, Shanghai, China) with protease inhibitor PMSF (Beyotime Institute of Biotechnology) and pulverized by ultrasonic wave on ice, followed by centrifugation at 13,400 ***g*** and 4°C. After that, protein content in the liquid supernatant was determined using a BCA Protein Assay Kit (ComWin Biotech Co., Ltd, Beijing, China) and 30 μg of protein from each sample were loaded onto 6–10% SDS-PAGE (Ten ovaries were used in each sample.). The proteins of interest were separated by electrophoresis, and transferred to PVDF (0.22 μm) membranes. The membranes were then blocked in 5% bovine serum albumin solution (Gibco BRL, dissolved in Tris-buffered saline containing 0.1% Tween 20) for 1 h and probed with specific primary antibodies overnight at 4 °C. Primary antibodies against ERK1/2(1:1000, 4695, CST, Boston, MA, USA), phosphor- ERK1/2 (Thr202/Tyr204) (1:1000, 4370, CST), mTOR (1:2000, 2972, CST), phosphor-mTOR (Ser2481) (1:1000, 2974, CST), RPS6(1:2000, 2217, CST), phosphor-RPS6 (Ser235/236) (1:2000, 2211,CST), AKT (1:1000, 4691, CST), phosphor-AKT (Ser473) (1:1000, 2211, CST), Forkhead box O3 (FOXO3A) (1:1000, 12829, CST), phosphor-FOXO3A (Ser253) (1:1000, ab47285, Abcam), PCNA (1:1000, ab152112, Abcam) and β-actin (1:10,000, 1970, CST) are all rabbit antibodies. HRP-conjugated goat anti-rabbit IgG (1:5000, 133997, Jackson ImmunoResearch Laboratories Inc.) was used to determine the proteins and an ECL kit (Millipore) was employed for visualization. ImageJ (National Institutes of Health, Java image processing software) was used to quantify the integrated light intensity of each band and to determine the concentration of phosphoproteins as well as the changes induced by treatment and calculate the ratio of phosphorylated proteins to their non-phosphorylated forms. β-actin expression was used as an internal control.

### Real-time quantitative RT-PCR (qPCR)

First, total RNA was isolated from ovaries using RNA prep pure Micro Kit (Qiagen) in accordance with the manufacturer’s protocols. RNA concentration was then measured with NANODROP 2000 (Thermo Scientific). Next, the extracted RNA (100 ng each for 20 µL reaction) was reverse-transcribed into cDNA using Transcriptor First Strand cDNA Synthesis Kit (Roche) according to the manufacturer’s instructions. Finally, the reverse transcription products were used as a template for PCR amplification with SYBR Premix Ex Taq II (TaKaRa). The following primer sequences were used: *GAPDH*: forward 5′-GGATGGAATTGTGAGGGAGA-3′ and reverse 5′-GTGGACCTCATGGCCTACAT-3′; *Sohlh1*: forward 5′-GAGAGAACGCAGGAGGAGGA-3′ and reverse 5′-GACATCTCCAGGACGGAAGC-3′; *Amh*: forward 5′-TGGCTGAAGTGATATGGGAGC-3′ and reverse 5′-TAGCACCAAATAGCGGGTGTC-3′. Each reaction well in a 96-well unclear plate (Axygen, San Francisco, CA, USA) contained 0.4  pmol/μL forward and reverse primers, 1 × HotstartFluo-PCR mix (TaKaRa) and 10 ng cDNA. The final reaction volume was 20 µL and all samples were run in triplicates. The amplification of cDNA was performed under the following conditions: pre-denaturation at 95°C for 30 s, followed by 40 cycles of denaturation at 95°C for 3 s and annealing at 60°C for 30 s in LightCycler 480 II (Roche). Data were collected and used for relative quantitative analysis with the 2^−ΔΔCT^ method. Relative mRNA expression level was obtained by comparing data of the experimental group with those of the control group and gene expression was normalized to *Gapdh*. The experiment was done in triplicate.

### Statistical analysis

All experiments were carried out in triplicates. Statistical analyses were performed using IBM SPSS Statistics for Windows, version 22.0 (IBM Corp.). Data were expressed as means ± s.d. and analysed by one-way ANOVA or non-parametric equivalent tests. Turkey test was used to compare two groups within three or four treatments.* P* < 0.05 was considered statistically significant.

## Results

### The expression of VIP and its receptor in the 4-day-old rat ovary

**Figure 1:**
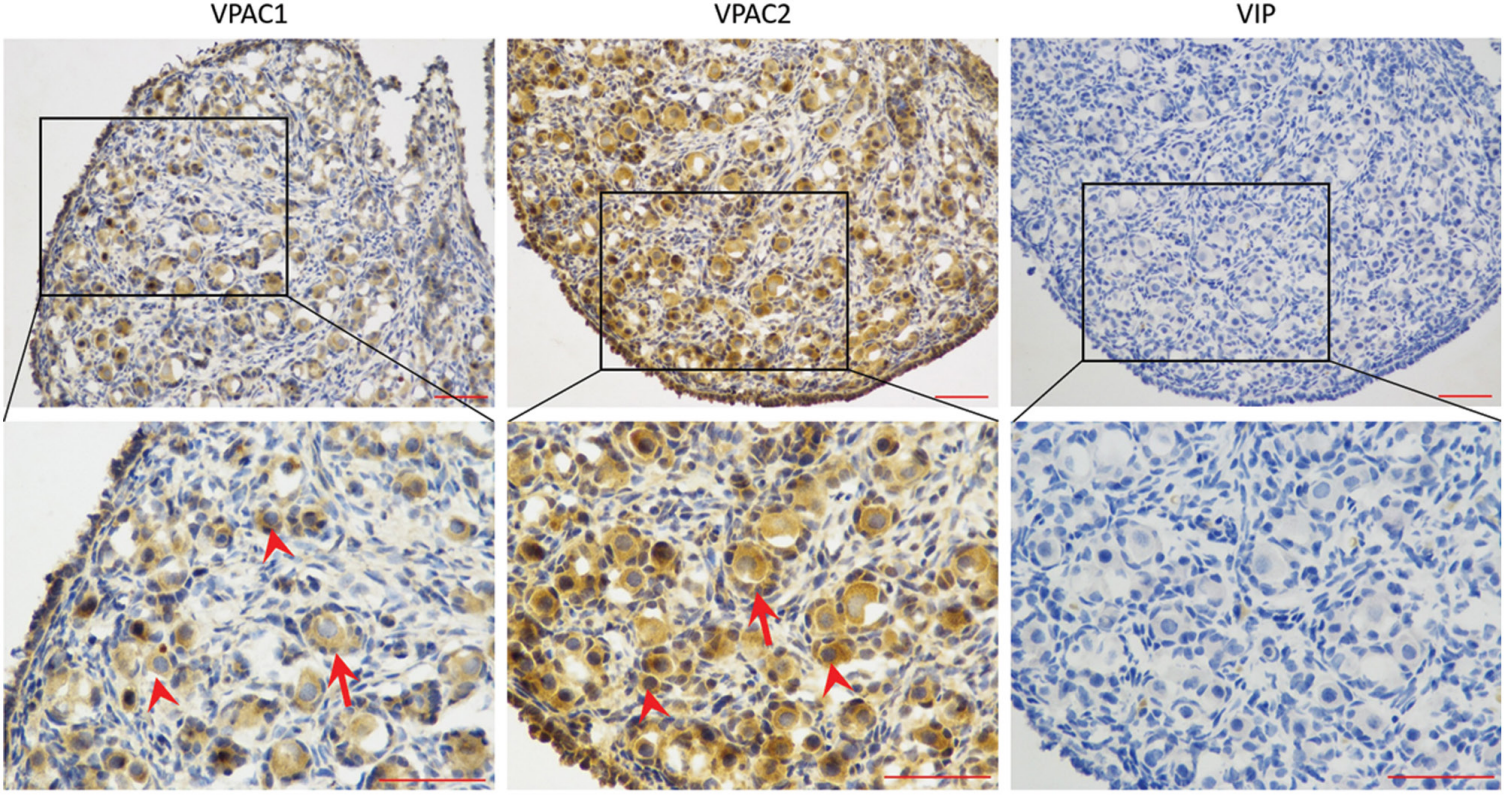
The expression of vasoactive intestinal peptide (VIP) and its receptors in 4-day-old rat ovary. Four-day-old rat ovaries were freshly collected and cut into 5 μm sections, followed by immunohistochemistry for detecting VIP and its receptors – VPAC1 and VPAC2 (arrowheads, positive primordial follicle; arrows, positive early growing follicles). Scale bar: 50 μm.

VIP takes effect mainly via its receptors 1 and 2 (VPAC1 and VPAC2), so we first examined whether these two receptors exist in the neonatal rat ovary by using anti-VPAC1 or VPAC2 antibody. IHC results showed that VPAC1 was mainly expressed in the cell membrane of granular cells and oocytes both in primordial and early-growing follicles, without positive stain in the ovarian cell nuclei or stroma. The expression pattern of VPAC2 was similar to that of VPAC1. There was no positive stain for VIP (Fig. 1). The specificity of the VPAC antibodies was confirmed by Western blot (Supplementary Fig. 1, see section on Supplementary data given at the end of this article).

### The effect of VIP on the activation of primordial follicle in neonatal rat ovary

**Figure 2:**
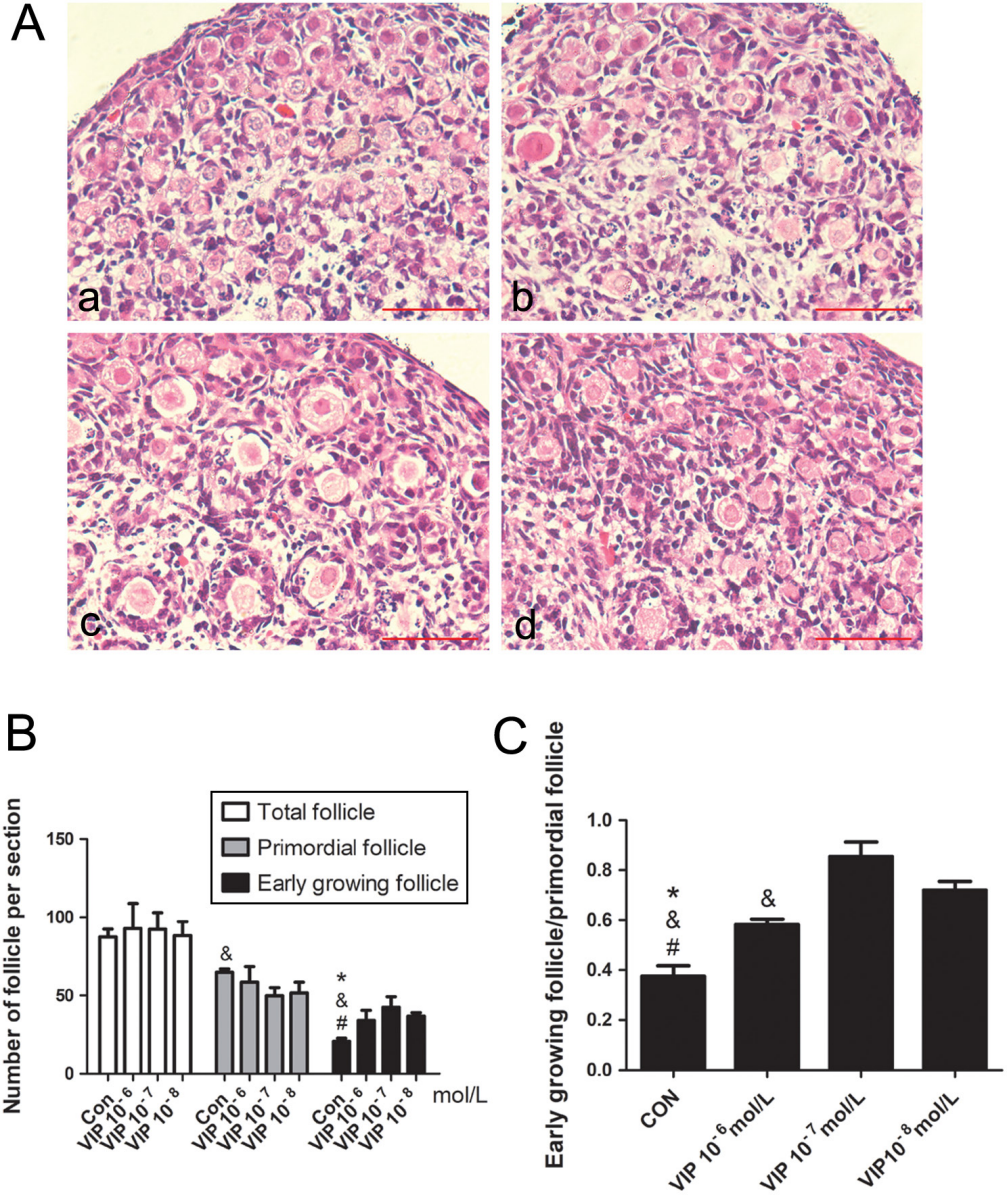
VIP promoted the transformation from primordial follicles to early growing follicles in neonatal rat ovary. Four-day-old rat ovaries were cultured in basal medium without VIP (control group) or with VIP in different concentrations for three days. (A) The representative photos of all groups stained with hematoxylin and eosin: control (a), 10^−6^ mol/L (b), 10^−7^ mol/L (c), 10^−8^ mol/L (d). (B) and (C) The numbers of early-growing follicles and primordial follicles were counted, and their ratio was calculated. Scale bar: 50 μm. Values are mean ± s.d. of at least three experiments. (*P < 0.05 compared with the VIP^−6^ mol/L group, ^&^ *P* < 0.05 compared with the VIP^−7^ mol/L group, ^#^ *P* < 0.05 compared with the VIP^−8^ mol/L group).

**Figure 3:**
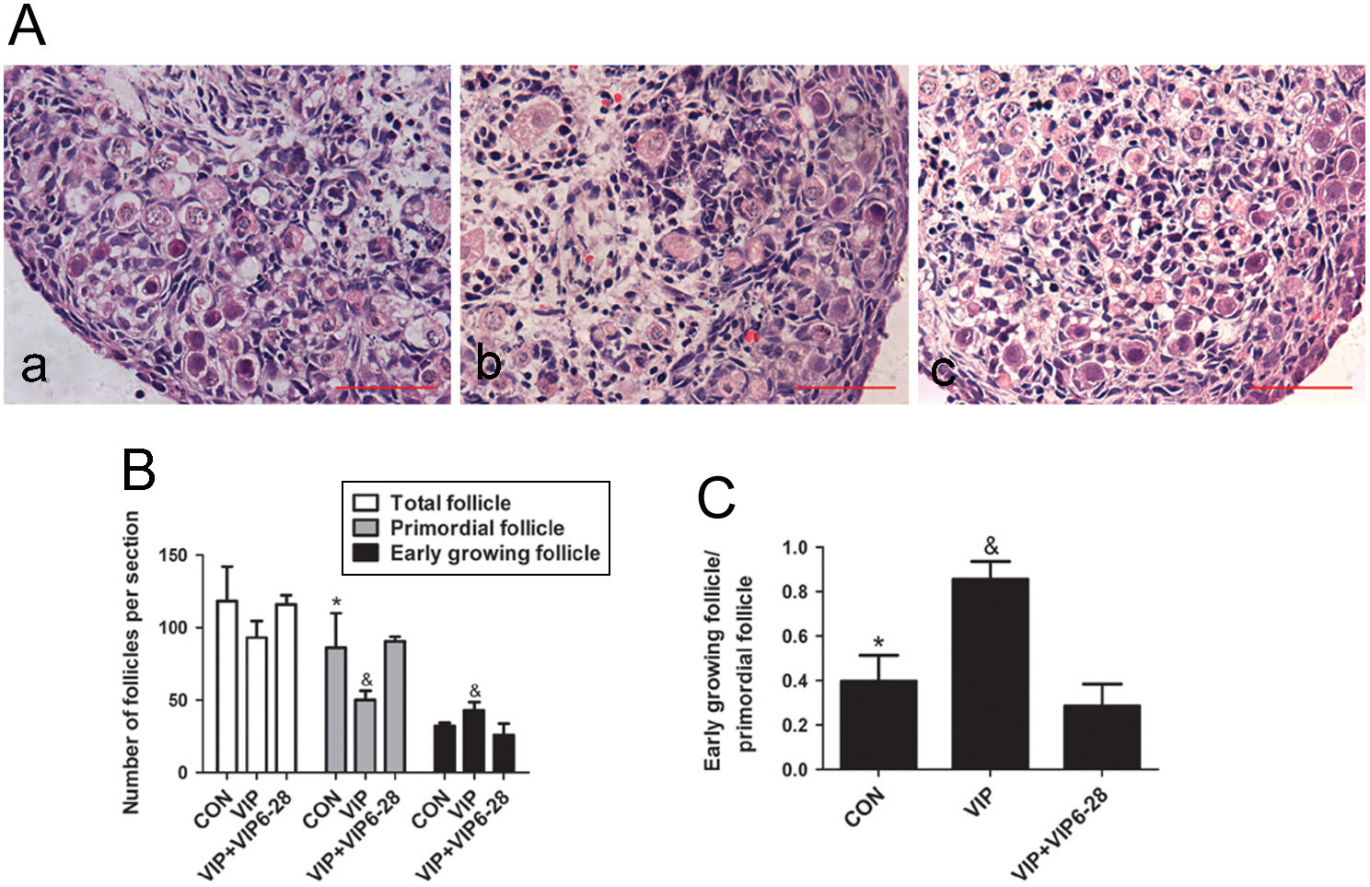
VIP_6–28_ inhibits the activation of neonatal rat ovary by VIP. Four-day-old rat ovaries were cultured in basal medium without VIP (control group) or with 10^−7^ mol/L VIP or VIP + VIP_6–28_ (5 × 10^−6^ mol/L) for 3 days. (A) The representative photos of all groups stained with hematoxylin and eosin: control (a), VIP (b), VIP+VIP_6–28_ (c). (B and C) The numbers of early-growing follicles and primordial follicles were counted, and their ratio was calculated. Scale bar: 50 μm. Values are mean ± s.d. of at least three experiments. (*P < 0.05 compared with the VIP-treated group, ^&^ *P* < 0.05 compared with the VIP+VIP_6–28_-treated group).

To explore the effects of VIP on follicle development and to determine the suitable concentration for an *in vitro* ovarian culture system, we cultured the 4-day-old ovaries for 3 days with different concentrations of VIP (10^−6^ mol/L, 10^−7^ mol/L and 10^−8^ mol/L). Hematoxylin and eosin (H&E) analysis showed that there were more early-growing follicles and less primordial follicles in the VIP-treated ovaries (Fig. 2A and B). The ratios of early-growing follicle/primordial follicle (GF/PF) in VIP-treated groups were higher compared with those of the control group (base culture medium without VIP), especially for the 10^−7^ mol/L VIP-treated group (Fig. 2C). To prove the specificity of VIP, ovaries were pre-treated with 5 × 10^−6^ mol/L VIP_6–28_, the specific antagonist of VIP receptors, for 2 h, and then co-treated with VIP and VIP_6–28_ for 3 days. In the VIP + VIP_6–28_ group, HE analysis showed less early-growing follicles as compared with the VIP-treated group (Fig. 3A and B); the ratio of GF/PF was similar to the control groups, which dropped to nearly half of that in the VIP-treated group (Fig. 3C). This outcome indicates that the activation of primordial follicle by VIP could be inhibited by VIP_6–28_ and that VIP acted specifically through its receptors VPAC1 and VPAC2 in the ovary.

**Figure 4:**
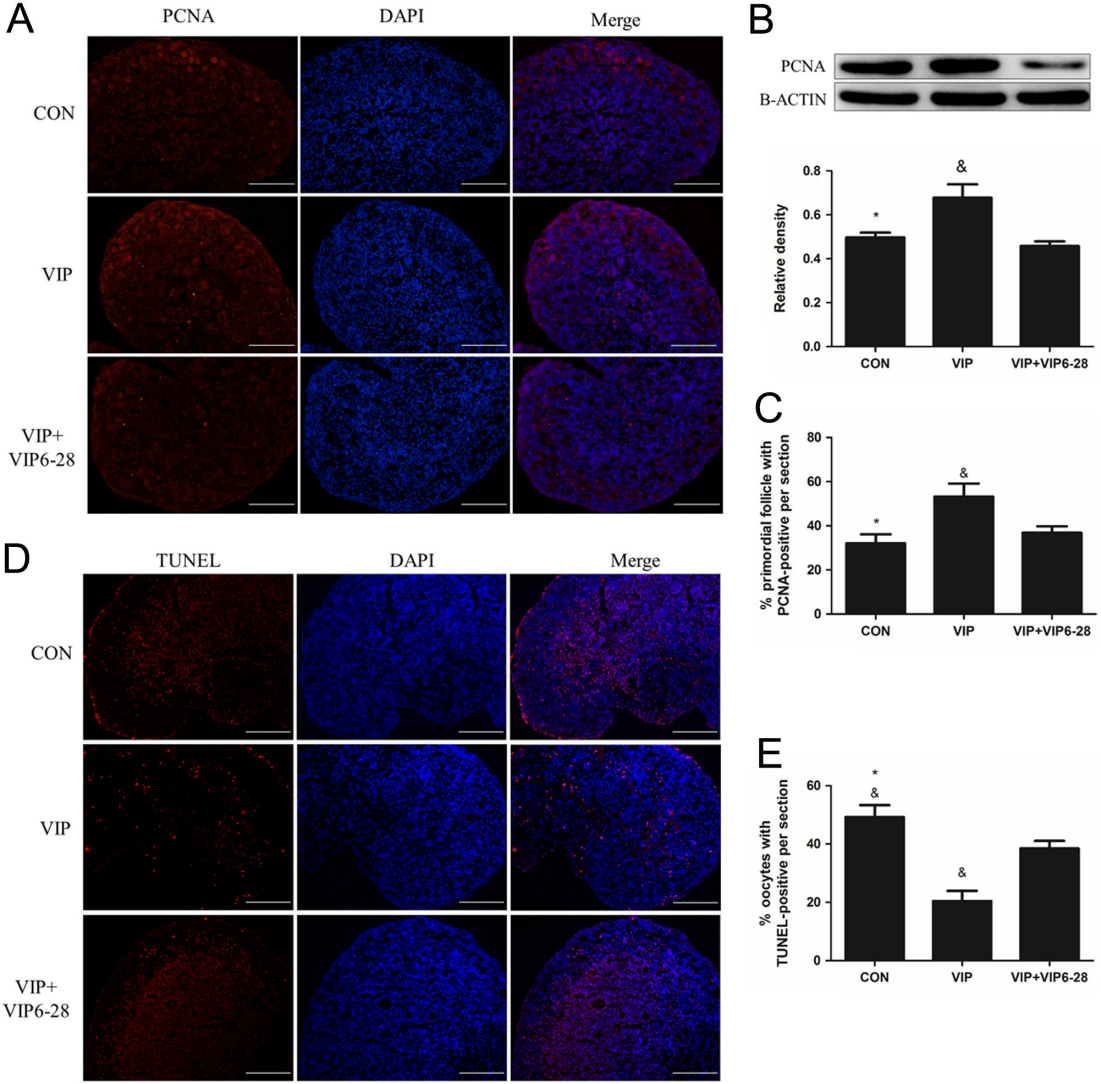
VIP promotes the proliferation of granular cells and inhibits the apoptosis of oocytes. Four-day-old ovaries were cultured with 10^−7^ mol/L VIP or VIP + VIP_6–28_ (5 × 10^−6^ mol/L) for 3 days. (A and D) PCNA staining and TUNEL assay were conducted in the three groups mentioned above for proliferation and apoptosis observation. (B) Western blot was conducted to quantify the expression of PCNA. (C and E) The percentage of primordial follicles or oocytes with PCNA-positive or TUNEL-positive section. Scale bar: 100 μm. Values are mean ± s.d. of at least three experiments. (*P < 0.05 compared with the VIP-treated group, ^&^ *P* < 0.05 compared with the VIP + VIP_6–28_-treated group).

As the activation of primordial follicles includes granular cell proliferation and oocyte growth (Zhang & Liu 2015), we then examined the effect of VIP on granular cell proliferation through proliferating cell nuclear antigen (PCNA) analysis and examined oocyte apoptosis using TUNEL assay. Compared to the control group or VIP + VIP_6–28_ group, more primordial follicles were observed to be PCNA-positive per section in the VIP group (53.2% ± 5.9% vs 32.0% ± 4.0%, 36.9 ± 2.9%, respectively). Result from Western blot also showed that VIP treatment increased the expression of PCNA by 1.5-fold compared to that of the other two groups (Fig. 4A, B and C). The ratio of apoptotic oocytes per section was 20.4 ± 3.5% in the VIP group, which is lower than the rest (49.2 ± 4.1%, 38.5 ± 2.6%) (Fig. 4D and E).

**Figure 5:**
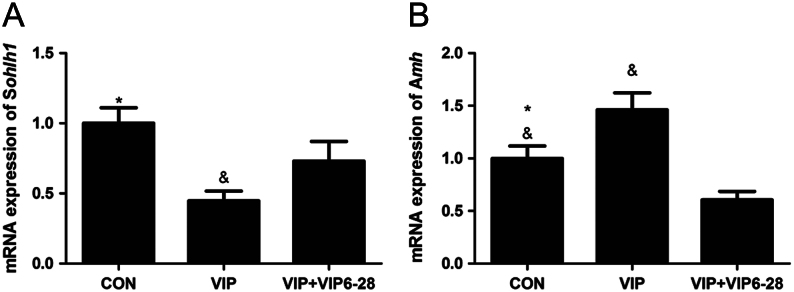
(A and B) The mRNA expression of *Sohlh1* and *Amh* in neonatal ovary after treatment with10^−7^ mol/LVIP with or without VIP_6–28_ (5 × 10^−6^ mol/L) for 3 days. Values are mean ± s.d. of at least three experiments. (Gene expression was normalized to *Gapdh*. *P < 0.05 compared with the VIP group, ^&^ *P* < 0.05 compared with the VIP + VIP_6–28_-treated group).

*Spermatogenesis and oogenesis-specific basic helix-loop-helix 1* (*Sohlh1*), a germ cell-specific gene that regulates the activation of primordial follicles, is mainly expressed in germ cell cysts and oocytes of primordial follicles in the ovary (Pangas *et al.* 2006, Jagarlamudi & Rajkovic 2012), while anti-Mullerian hormone (*Amh*) (Dayal *et al.* 2014), the key mediator of early follicular differentiation, is expressed in granular cells of growing follicles. We thus examined the mRNA expression of *Sohlh1* and *Amh* to evaluate the activation of primordial follicles. Four-day-old rat ovaries were treated with 10^−7^ mol/L VIP with or without VIP_6–28_ for 3 days, followed by RNA isolation and qRT-PCR. Results indicated that the mRNA expression of *Sohlh1* was significantly decreased in the VIP-treated group but increased with the addition of VIP_6–28_ (Fig. 5A). Meanwhile, the mRNA expression of *Amh* increased in the VIP-treated group but decreased in the VIP + VIP_6–28_-treated group (Fig. 5B), indicating that VIP is indeed able to promote the development of primordial follicles into growing follicles.

### VIP activates primordial follicles through the ERK-mTOR signalling pathway

**Figure 6:**
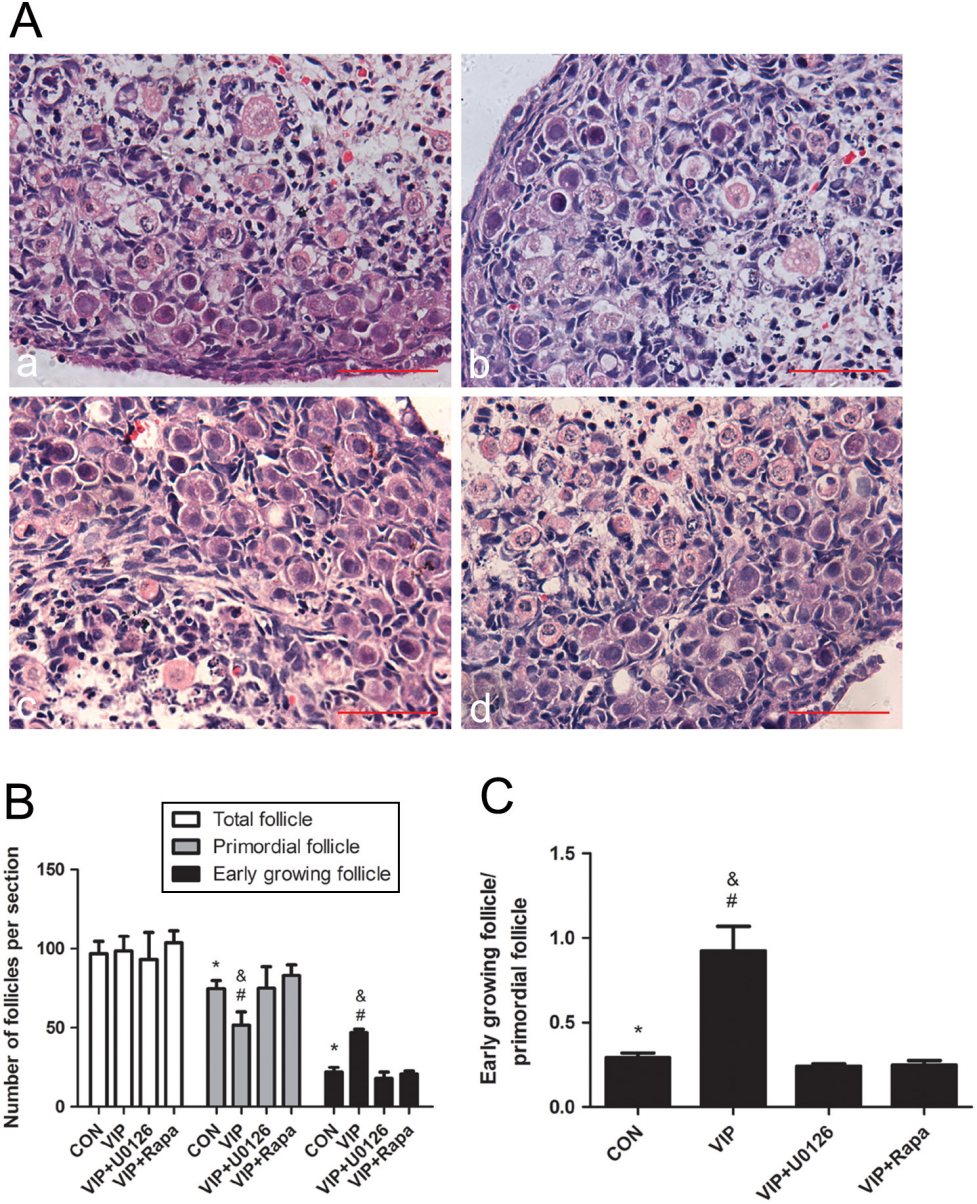
Effect of U0126 or rapamycin on VIP-induced follicle development. Four-day-old rat ovaries were cultured with (i) 10^−7^ mol/L VIP, (ii)VIP plus 10^−8^ mol/L U0126, or (iii) VIP + 8.75×10^−8^ mol/L rapamycin for three days. (A) HE stained ovaries of different groups: control (a), VIP (b), VIP + U0126 (c), VIP + Rapa (d). (B and C) The numbers of early-growing follicles and primordial follicles were counted, and their ratio was calculated. Scale bar: 50 μm. Values are mean ± s.d. of at least three experiments. (*P < 0.05 compared with the VIP group, ^&^ *P* < 0.05 compared with the VIP + U0126 group, ^#^ *P* < 0.05 compared with the VIP + Rapa group).

**Figure 7:**
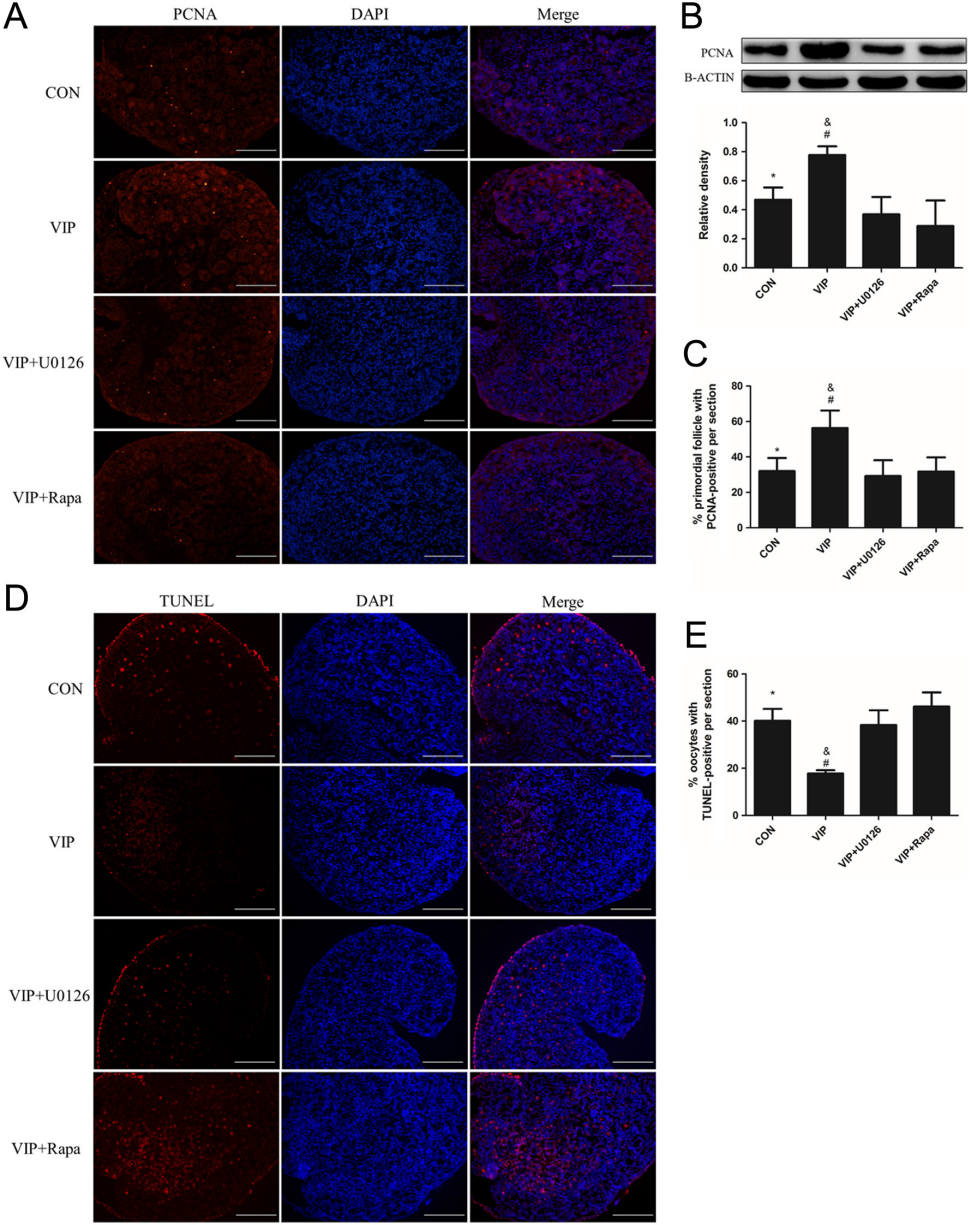
U0126 or rapamycin inhibits the VIP-induced proliferation of granular cells and promotes oocyte apoptosis. Ovaries were cultured with (i) 10^−7^ mol/L VIP, (ii) VIP + 10^−8^ mol/L U0126 or (iii) VIP plus 8.75 × 10^−8^ mol/L rapamycin for 3 days. (A and D) PCNA staining and TUNEL assay were conducted for proliferation and apoptosis observation. (B) Western blot was conducted to quantify the expression of PCNA. (C and E) The numbers of primordial follicles with positive PCNA staining and oocytes with positive TUNEL staining were counted. Scale bar: 100 μm. Values are mean ± s.d. of at least three experiments. (*P < 0.05 compared with the VIP group, ^&^ *P* < 0.05 compared with the VIP + U0126 group, ^#^ *P* < 0.05 compared with the VIP + Rapa group).

To explore the possible mechanism by which VIP activates neonatal rat primordial follicles, ovaries were cultured for 3 days with VIP or VIP plus 10^−8^ mol/L U0126 (VIP + U0126), which inhibits the phosphorylation of MEK or VIP plus 8.75 × 10^−8^ mol/L rapamycin (VIP + Rapa), the inhibitor of mTORC1. As seen in Fig. 6, as indicated in H&E staining, treatment with either U0126 or rapamycin could decrease the ratio of GF/PF as compared to that in the VIP-treated groups. The number of PCNA primordial-positive follicles and the expression of PCNA were also decreased as quantified by western blot (Fig. 7A, B and C). Meanwhile, TUNEL assay result showed that the occurrence of apoptosis increased with the presence of any of the two inhibitors (Fig. 7D and E).

**Figure 8:**
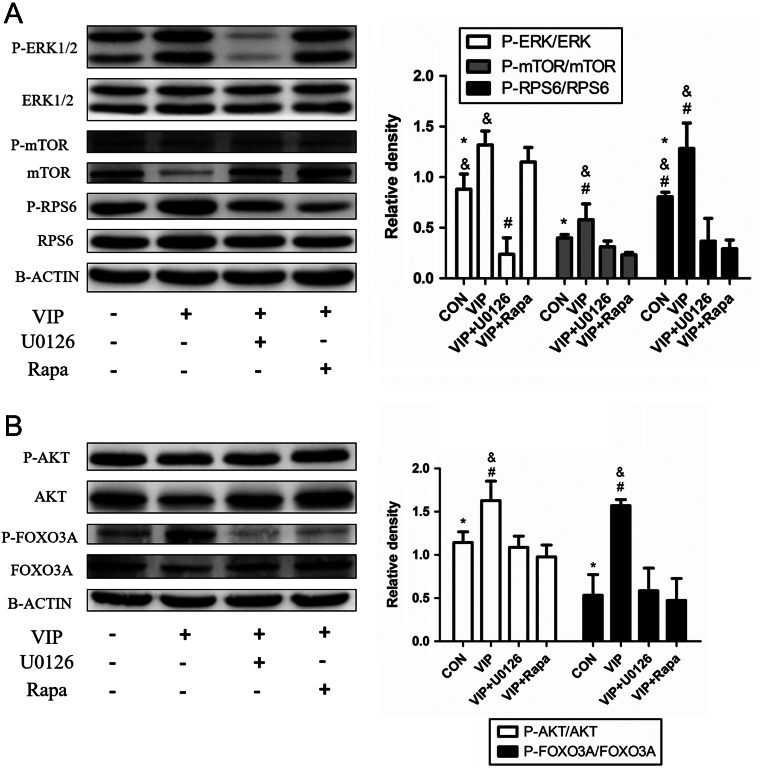
VIP activated primordial follicle through ERK-mTOR signalling way. Ovaries were cultured with 10^−7^ mol/L VIP + 10^−8^ mol/L U0126 or 8.75 × 10^−8^ mol/L rapamycin for 3 days. (A and B) Western blot was conducted to investigate the level of protein phosphorylation in the ERK-mTOR signalling pathway, as well as AKT and FOXO3A. Values are mean ± s.d. of at least three experiments. (*P < 0.05 compared with the VIP group. ^&^ *P* < 0.05 compared with the VIP + U0126 group. ^#^ *P* < 0.05 compared with the VIP + Rapa group).

The phosphorylation of ERK1/2 in the VIP groups significantly increased compared with that in the control group and the VIP + U0126 group but was similar to that in the VIP + Rapa groups. On the other hand, the phosphorylation of mTOR and its downstream protein, RPS6, were significantly increased compared to that in the other groups (Fig. 8B).

It is well known that primordial follicle initiation is accompanied by the activation of the phosphatidyinositol-3-kinase (PI3K)/AKT/FOXO3Asignaling pathway in oocytes (Zhang & Liu 2015). Hence, we examined the expression of AKT and FOXO3A phosphorylation. Result indicated that the phosphorylation of AKT and FOXO3A were significantly decreased in the VIP+U0126 and VIP + Rapa groups, reflecting less primordial follicle activation (Fig. 8A).

## Discussion

Although much research has been done on the mechanism of follicular activation, the specific mechanism of primordial follicle initiation is still not completely understood. Our study has demonstrated the ability of VIP in promoting primordial follicle activation through the ERK-mTOR signalling pathway in neonatal rat ovary, providing evidence and insight into the effect and mechanism of the microenvironment that is involved in the activation of primordial follicle.

Both VIP and its receptors had been found in preantral and antral follicles in the ovaries of several mammals. The expression of VIP is increased during luteinizing hormone surge in granular cells and follicular fluid. The mRNA transcripts for VIP receptors were identified in 22-day-old mice ovaries and in ovarian follicles at different developmental stages of pigs, as well as in ovarian extracts of human foetuses and women (Barberi *et al.* 2007, Gabbay-Benziv *et al.* 2012, Bukowski & Wąsowicz 2015). As for the proteins, VPAC1 was identified in follicles, mostly in the oocytes, of all human foetus samples (22 gestational weeks onward) and some samples from women and girls (Gabbay-Benziv *et al.* 2012). However, only a minority of the samples from all three sources contained follicular VPAC2 protein. Nonetheless, a number of other researches had stated that VPAC2 was in fact more widespread than VAPC1, with positive stain observed in nearly all tissues (Harmar *et al.* 2012). In our study, we demonstrated that the proteins for VIP receptors do exist in primordial follicles and in early-growing follicles of 4-day-old rat ovaries, both in the granular cells and oocytes, which is similar to the other mammals mentioned earlier. All these findings suggest that VIP may be involved in the growth and development of follicles. Although the staining of protein for VIP yielded negative result, we had since confirmed the presence of VIP mRNA in rat ovaries (data not shown). A previous study showed similar outcome whereby protein staining for the VIP system yielded weak result, especially in foetal samples (Gabbay-Benziv *et al.* 2012). We speculate that this may be due to the rapid degradation of the VIP peptide.

It was known that VIP could cause a dose-dependent increment in aromatase activity and in levels of cAMP prior to folliculogenesis (George & Ojeda 1987), which is related to the development of primordial follicles. This relationship suggests the probable role of VIP in promoting growth and development of early follicles. Mayerhofer *et al.* have reported that the mRNA transcript of FSH receptor was increased in 2-day-old rat ovaries that were cultured *in vitro* for 8 h with VIP. Follicular growth was then increased when rat ovaries were exposed to FSH (Mayerhofer *et al.* 1997). Since preantral follicles do not react with FSH, they would have possibly been promoted into antral follicles which are sensitive to FSH with the assistance of VIP. In addition, Bruno *et al.* also highlighted that VIP could improve the survival and development of caprine preantral follicles after *in vitro* tissue culture (Bruno *et al.* 2010), illustrating its possible crucial role in early follicle development and improving the quality of eggs that mature *in vitro*. Our study proves that VIP could promote the activation of primordial follicles in neonatal rat ovaries, induce granular cells proliferation and inhibit oocytes apoptosis. Furthermore, these effects were inhibited by VIP_6–28_, the antagonist of VIP receptors, leading to the conclusion that VIP takes effect in neonatal ovaries via its specific receptors.

There are few reports about the mechanism by which VIP affects the development of follicles. We have demonstrated that VIP activates neonatal primordial follicle of rat through the ERK-mTOR signalling pathway. *In vivo*, VIP mainly acts through the cAMP–PKA pathway after binding to its specific receptors on cell membranes. Many proteins (including MAPK kinase) can be phosphorylated after PKA activation, resulting in a series of physiological effects, especially in the proliferation and differentiation of cells (Couvineau & Laburthe 2012). It has been shown that VIP could mediate the proliferation of rat brain microvascular endothelial cells through the cAMP-PKA signalling pathway, supporting the claim mentioned earlier (Yang *et al.* 2013). Moreover, VIP increases the level of cAMP prior to folliculogenesis, while ERK and mTOR have been proven to be essential for primordial follicular activation. Therefore, our result is consistent with the findings of previous reports. Zhang *et al.* proved that the phosphorylation of mTOR in pregranular cells was the primary step for follicle recruitment in gene-knockout mice (Zhang *et al.* 2014). As VPAC exists in granular cell and oocytes, VIP may affect both during the activation of primordial follicles.

It is universally acknowledged that the PI3K–PTEN (TENsin homology deleted in chromosome 10)-AKT-FOXO3 signalling pathway participates in the recruitment of primordial follicles in mammals, which governs the activation of oocyte in primordial follicles (Zhang & Liu 2015). In this study, we have shown that the phosphorylation of AKT and FOXO3A was significantly increased. This reflects the possibility of oocyte activation followed by the activation of pre-granular cells or the direct effect of VIP on oocytes. However, some researches proposed that mTOR is located downstream of AKT in primordial follicle activation (Saxton & Sabatini 2017), which is contradictory of the Western blot result in this study whereby the addition of rapamycin was able to decrease the phosphorylation of AKT. According to the study in gene-knockout mice by Zhang *et al.*, it is possible that VIP first activates the mTOR signalling pathway in pre-granular cells, which subsequently leads to the activation of PI3K/AKT signalling pathway in oocyte (Zhang *et al.* 2014).

Some researchers had attempted to use stimulators of AKT or PI3K combined with inhibitor of PTEN to activate dormant follicles in the treatment of patients with primary ovarian insufficiency as part of the IVA strategy (Suzuki *et al.* 2015, Zhai *et al.* 2016). Although follicle growth appeared in some patients, successful pregnancies were rare, only two in 37 patients or one in 14 patients. The PTEN inhibition could result in increased DNA damage and impaired DNA repair capacity in ovarian follicles while promoting bovine non-growing follicle activation (Maidarti *et al.* 2019). We do acknowledge that the activation of primordial follicles is indeed complex. The PI3K-PTEN-AKT-FOXO3 or ERK-mTOR or other signalling pathways may only be a small component of the full mechanism which is yet to be fully elucidated. Thus, it is difficult to acquire full success by relying only on stimulating those signalling pathways. There are proposals that the microenvironment and the local factors surrounding individual primordial follicle are likely to play defining roles in deciding the fates of these follicles as well, since many growth factors have been reported to be functional in regulating the activation of primordial follicles *in vitro* (Zhang & Liu 2015). Therefore, the success rate of IVA might be improved through the right combination of useful growth factors and stimulators. Moreover, considering the risk of ovarian tissue auto-replantation in cancer patients, it will be beneficial if mature oocytes could be acquired *in vitro*. It is necessary to study the microenvironment as IVA is of great importance as the first step to treating infertility. VIP may emerge as one of the growth factors that could be used in IVA to obtain matured oocytes *in vitro*.

Last but not least, the *in vitro* culture system in our study may not be sufficient for healthy follicle development, since quite a few early-growing follicles appeared to be disordered in terms of granulosa cell arrangement, especially in VIP-treated ovaries. It might be due to lack of necessary factors, like leukaemia inhibitory factor, basic fibroblast growth factor and platelet-derived growth factor, which had been reported to promote follicle development (Zhang & Liu 2015). Besides, there were more early-growing follicles that were activated by VIP than primordial follicles. Therefore, it is reasonable for a larger number of healthy oocytes to be observed in the control group as compared with that in VIP-treated group. Other necessary factors must be taken into account for the application of VIP in IVA to stimulate and retain healthy follicles.

## Conclusion

This study demonstrated the ability of VIP to activate primordial follicles of neonatal rat ovary *in vitro*, likely through the ERK–mTOR signalling pathway. Our findings provide a new strategy for *in vitro* activation of dormant follicles.

## Supplementary Material

Supplementary Figure 1

## Declaration of interest

The authors declare that there is no conflict of interest that could be perceived as prejudicing the impartiality of the research reported.

## Funding

This work was supported by grants from Sun Yat-Sen University Clinical Research 5010 Program (2006004), from Sun Yat-Sen Clinical Research Cultivating Program, Guangdong Science and Technology Department (2017B030314026).

## Author contribution statement

Song Li, Qi Fan and Qingxue Zhang contributed significantly to the conception and design of this work. Haiyan Lin and Qi Qiu contributed to data analyses. Song Li, Yanqiu Xie and Yihua Liang wrote the manuscript. All authors revised the paper and approved the final version.
