# Supplementary figures and images for: VIP activates primordial follicles of rat through ERK-mTOR pathway in tissue culture
Source: Reproduction. 2019 Feb 26;157(5):475–84. doi: 10.1530/REP-18-0466 (PMC6433003; doi:10.1530/REP-18-0466)

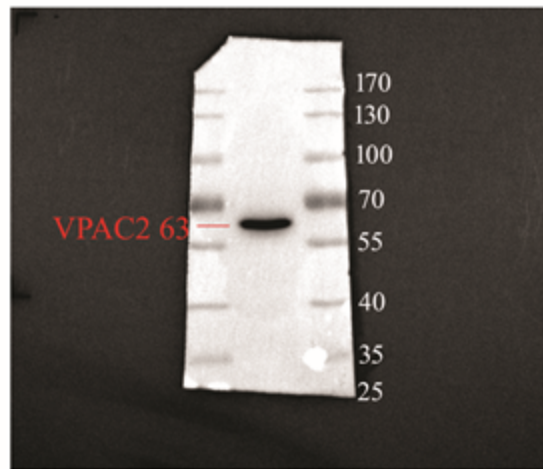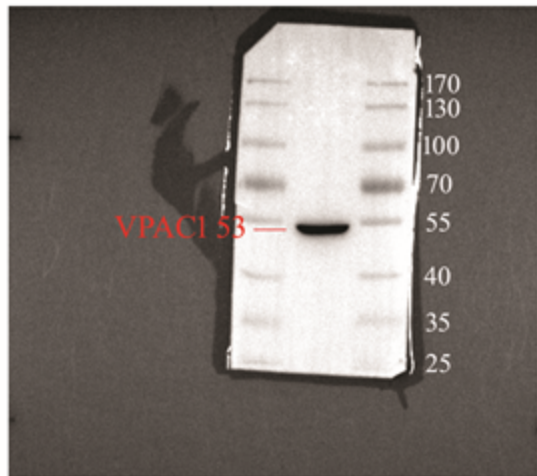

Supplemental figure. The western blot showing specificity of antibodies for VPAC1 and VPAC2.

Supplement: Supplementary Figure 1 [file supplementary_figure_1.pdf]
